# A procephalic territory in *Drosophila *exhibiting similarities and dissimilarities compared to the vertebrate midbrain/hindbrain boundary region

**DOI:** 10.1186/1749-8104-2-23

**Published:** 2007-11-05

**Authors:** Rolf Urbach

**Affiliations:** 1 Institute of Genetics, University of Mainz, Johannes-Joachim Becherweg 32, Mainz, Germany, D-55128

## Abstract

**Background:**

In vertebrates, the primordium of the brain is subdivided by the expression of Otx genes (forebrain/anterior midbrain), Hox genes (posterior hindbrain), and the genes Pax2, Pax5 and Pax8 (intervening region). The latter includes the midbrain/hindbrain boundary (MHB), which acts as a key organizer during brain patterning. Recent studies in *Drosophila* revealed that orthologous sets of genes are expressed in a similar tripartite pattern in the late embryonic brain, which suggested correspondence between the *Drosophila* deutocerebral/tritocerebral boundary region and the vertebrate MHB. To gain more insight into the evolution of brain regions, and particularly the MHB, I examined the expression of a comprehensive array of MHB-specific gene orthologs in the procephalic neuroectoderm and in individually identified neuroblasts during early embryonic stages 8–11, at which the segmental organization of the brain is most clearly displayed.

**Results and conclusion:**

I show that the early embryonic brain exhibits an anterior *Otx*/*otd *domain and a posterior *Hox1*/*lab *domain, but that *Pax2*/*5*/*8 *orthologs are not expressed in the neuroectoderm and neuroblasts of the intervening territory. Furthermore, the expression domains of *Otx*/*otd *and *Gbx*/*unpg *exhibit a small common interface within the anterior deutocerebrum. In contrast to vertebrates, *Fgf8*-related genes are not expressed posterior to the *otd*/*unpg *interface. However, at the *otd*/*unpg *interface the early expression of other MHB-specific genes (including *btd*, *wg*, *en*), and of dorsoventral patterning genes, closely resembles the situation at the vertebrate MHB. Altogether, these results suggest the existence of an ancestral territory within the primordium of the deutocerebrum and adjacent protocerebrum, which might be the evolutionary equivalent of the region of the vertebrate MHB. However, lack of expression of *Pax2*/*5*/*8 *and *Fgf8*-related genes, and significant differences in the expression onset of other key regulators at the *otd*/*unpg *interface, imply that genetic interactions crucial for the vertebrate organizer activity are absent in the early embryonic brain of *Drosophila*.

## Background

In vertebrates, the primordium of the brain is subdivided along the anteroposterior (AP) axis into three basic regions, reflected by the restricted expression of a highly conserved set of developmental genes before these brain regions become morphologically distinct. *Otx *genes are expressed in the anterior region, which comprises the forebrain and anterior midbrain, *Hox *genes in the posterior region comprising the hindbrain, and the genes *Pax2*,*Pax5 *and *Pax8 *in the intervening region. The intervening region includes the territory of the midbrain/hindbrain boundary (MHB), which encompasses the posterior part of the midbrain and rhombomere 1 of the hindbrain. The position of the MHB is controlled by the interface between the expression domains of *Otx2 *and *Gbx2*. The MHB exerts organizer properties that play an essential role in patterning the midbrain and hindbrain [1,2]. These organizer activities are mediated by fibroblast growth factor 8 (Fgf8) and Wnt1 proteins, which are secreted from the MHB neuroectoderm. The MHB (or isthmic) organizer arises in consecutive developmental steps that are mirrored by the ordered temporal sequence of MHB-specific gene expression. Its development initiates with the formation of an *Otx2/Gbx2 *interface, where in a second step *btd/Sp-related 1 *(*Bts1*), *Pax2*, *Fgf8 *and *Wnt1 *become expressed. In a third step, in addition to the already activated genes, their downstream targets are upregulated, among which are *Pax5*, *Pax8*, *En1 *and *En2*, whose pathways are mutually dependent with respect to maintaining the boundary [1-5].

The brains of deuterostomes (for example, tunicates and vertebrates) and protostomes (for example, arthropods and annelids) both seem to contain a rostral domain specified by the *Otx/otd *family, and a caudal domain specified by genes of the *Hox *family (for example, reviewed by [6-10]). Expression of *Pax2/5/8 *in the intervening neck region between the *Otx *and *Hox1 *domains has been observed in vertebrates and in the closely related ascidian tunicates, suggesting that this tripartite ground pattern of the brain is conserved during evolution within the chordate lineage [11]. Moreover, in the ascidian *Ciona*, the expression and activation of other crucial MHB determinants in the neck region, such as of *Fgf8/17/18 *and *Engrailed *(*En*) orthologs, are reminiscent of those in vertebrates [12,13], suggesting that the conserved pattern of their expression also pre-dates the splitting of the vertebrates from the chordate lineage. However, since the expression of *Pax2/5/8 *and *En *is absent in the intervening neck of appendicularian tunicates [14], and the neck region of another invertebrate chordate, amphioxus, lacks expression of *Pax2/5/8*, *En *and *Wnt *[15-17], this has raised doubt about the existence of a MHB territory in invertebrate chordates (irrespective of whether it includes organizer properties or not) [18,19]. On the other hand, in the late embryonic brain of *Drosophila*, a tripartite pattern of *Otx*, *Pax2/5/8*, and *Hox1 *expression has been reported, with *Pax2/5/8 *expression located at the interface between the domains of *Otx/otd *and *Gbx/unpg*, and coinciding with the neuromeric border between deutocerebrum and tritocerebrum. These findings have led to the hypothesis that the *Drosophila *deutocerebral/tritocerebral boundary region and the vertebrate MHB are corresponding structures, and that a basic tripartite regionalization of the brain was existent already in the common ancestor of the bilaterians [20,21].

To broaden the perspective on the evolution of brain regions, and in particular the MHB, I have undertaken a comprehensive analysis of orthologous factors of vertebrate MHB-specific regulatory genes in the *Drosophila *early embryonic brain. Since the specification of the MHB is one of the earliest decisions in the developing vertebrate brain, taking place before and during the formation of neuroblasts, I focussed on the early period of embryonic brain development. I describe the expression of MHB-specific marker genes at a resolution of identified neuroblasts (NBs) and in relation to the segmental architecture of the brain at stages when it is most clearly displayed. Based on the expression of *orthodenticle *(*otd *(*oc*, Flybase)) and *labial *(*lab*), the early brain principally exhibits a tripartite pattern with an anterior *otd *domain, a posterior *Hox *(that is, *lab*) domain, and a territory intervening between both domains. However, the *Pax2/5/8 *orthologs, *D-pax2 *(*sv*, Flybase) and *pox neuro*, are not expressed in the neuroectoderm and brain NBs of the intervening territory. Moreover, I identified a small interface between the complementary procephalic domains of *otd *and *unplugged *(*unpg*) that is located within the anterior deutocerebrum, corresponding to the anterior border of the intervening zone. The expression of these and further MHB-specific genes (such as the *Wnt1 *ortholog *wingless*, the *En1*,*2 *ortholog *engrailed*, and the zebrafish *Bts1 *ortholog *buttonhead*), and of dorsoventral (DV) patterning genes (the *Msx *ortholog *muscle specific homeobox *(*msh *(*Dr*, FlyBase)) and the *Nkx2 *ortholog *ventral nervous system defective*) in relation to the *otd/unpg *interface suggests that the neuroectoderm around this interface may represent an ancestral territory, evolutionarily equivalent to the neuroectodermal region at the MHB in vertebrates. However, in this part of the early embryonic brain, the expression of other MHB-specific markers (the *Fgf8*-related genes, *branchless*, *pyramus*, and *thisbe*) exhibits profound differences compared to the embryonic MHB domain in vertebrates. This suggests that, for the initial period of neurogenesis, the expression and regulatory interactions of genes, and the accompanying functional properties of the neuroectodermal territory around this interface, have changed during evolution.

## Results

In vertebrates, the specification of the MHB is one of the earliest steps in brain development, taking place before and during the formation of NBs [4]. Therefore, in this comparative study in *Drosophila *I largely focussed on the early developmental period until embryonic stage 11, throughout which the pattern of NBs in the brain and ventral nerve cord (VNC) is fully established [22,23]. Furthermore, stage 11 represents the phylotypic stage of development [24] at which the segmental organization of the brain is most clearly displayed [25], and to which the expression patterns of MHB-specific gene orthologs can most accurately be related.

### Expression of Otd and Labial regionalizes the anlagen of the embryonic brain and demarcates an intervening zone

**Figure 1:**
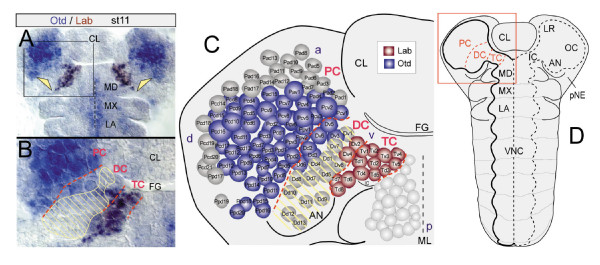
The brain anlagen in *Drosophila *is tripartite. **(a) **Flat preparation of the head of an Otd/Labial antibody double stained embryo at stage 11. **(b) **Close-up of the region framed in (a) at the level of brain NBs. **(c) **Schematic summary of the expression of both genes in identified brain NBs in the left hemisphere at late stage 11, corresponding to the boxed area in (d); red dashed lines indicate neuromeric boundaries. An intervening zone can be identified between the domains of Otd and Lab expression, in which both genes are expressed neither in the peripheral ectoderm (yellow arrowheads in (a)) nor in the deriving NBs (hatched area in (b,c)). Note that in the deutocerebrum (DC) some anterior NBs (Dv5,6, Dd2,3,6) express Otd, and some posterior NBs (Dv2,4) express Labial (NB nomenclature according to [23]). **(d) **Segmental topography of the *Drosophila *embryo at the phylotypic stage of development (stage 11); flat preparation (anterior to the top), the head capsule has been opened dorsally. The pregnathal (labral (LR), ocular (OC), antennal (AN), intercalary (IC)) and gnathal head segments are indicated on the right side. On the left side, the primordium of the CNS is outlined (PC, protocerebrum; DC, deutocerebrum; TC, tritocerebrum; MD, mandibular, MX, maxillary, and LA, labial neuromere, respectively). Abbreviations: a, d, p, v, anterior, dorsal, posterior, ventral; AN, antennal appendage; CL, clypeolabrum; FG, foregut; ML, midline; VNC, ventral nerve cord.

In vertebrates and the closely related invertebrate chordates (that is, urochordates or tunicates), the neuroectoderm exhibits a fundamental tripartite organization already at early developmental stages: *Otx *is expressed in an anterior domain, *Hox1 *in a posterior domain, and *Pax2/5/8 *in an intervening territory [18,19]. In the late embryonic brain of *Drosophila*, orthologous sets of genes have been shown to be expressed in a tripartite pattern as well [20]. To see if a comparable pattern of gene expression exists in the early anlagen of the *Drosophila *brain (procephalic neuroectoderm (pNE) and NBs), I investigated the expression of the orthologous genes *orthodenticle *(*otd*) and *labial *(*lab*). The domain of Otd expression covers the anterior part of the antennal and most of the ocular pNE, and is found in most NBs of the protocerebrum (PC) and some anterior NBs of the deutocerebrum (DC; see also [26]). The posterior border of Otd expression is positioned within the anterior DC (Figure 1a, b). The domain of Lab expression covers the pNE of the intercalary segment and all NBs of the tritocerebrum (TC) as well as two NBs of the DC (Dv2,4; see also [26]). The anterior limit of the Lab domain is tightly linked to the segmental border between TC and DC (Figure 1b).

Thus, the early embryonic *Drosophila *brain discloses an anterior Otd domain, a posterior *Hox *domain (that is, of Lab expression), and an 'intervening zone' (IZ) encompassing a fraction of deutocerebral NBs in which neither gene is expressed (Figure 1c).

### Expression of *Pax2/5/8 *orthologous genes is missing in the pNE and NBs of the intervening zone

**Figure 2:**
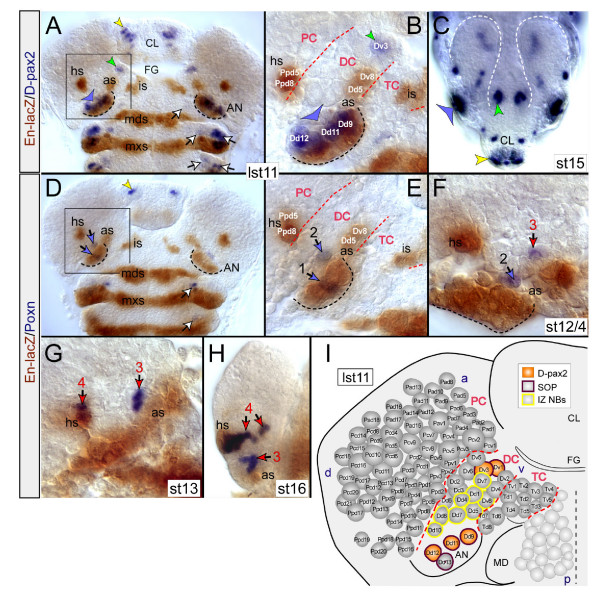
Expression of Poxn and D-pax2 is lacking in NBs of the IZ. (**a,b**) D-pax2/En-lacZ double stainings; flat preparations of late stage 11 (lst11) embryos. (b) Magnification of boxed area in (a) at the level of NBs. En-lacZ-positive NBs deriving from the antennal *en *stripe (as; Dv8, Dd5) and *en *head spot (hs; Ppd5,8) are indicated. D-pax2 is detected in SOPs of the dorsal organ (blue arrowhead), the hypopharyngeal/latero-hypopharyngeal organ (green arrowhead), and of the labral sensory organ (yellow arrowhead in (a)) [23]. Note that SOPs at positions corresponding to those of the dorsal organ within the developing antennal appendage (AN; outlined by black dashed line) are also found in the appendage of the mandibular and maxillary segment (white arrows in (a)). (**c**) D-pax2 stained whole mount embryo at stage 15 (st15) with a focus on the brain (outlined by the white dashed lines). D-pax2 is found in the respective sensory organs. (d-h) Poxn/En-lacZ double stained embryos. (**d**) Late stage 11 (lst11). Few Poxn-positive cells contribute to the labral (yellow arrowhead) and antennal (blue arrows) sensory organs. Poxn sensory founder cells arise at corresponding positions in the antennal, mandibular, maxillary, and labial segments (white arrows), immediately anterior to the respective *en *stripes. Note that in the CNS, Poxn expression is not yet initiated. (**e**) Close-up of boxed area in (d). Poxn expression initiates in peripheral ectodermal cells (group 1, 2) positioned in the antennal appendage, which are likely to contribute to the antennal dorsal organ. (**f**) By stage 12/4, Poxn expression comes up in cells of the DC (group 3) immediately anterior to the antennal *en *stripe (as). (**g**) By stage 13, first *en*-coexpressing cells (group 4), descending from the two head spot (hs) NBs, initiate Poxn expression. Note that the head spot NBs are Poxn-negative. (**h**) Stage 16. The number of Poxn-positive cells in the deuto- and protocerebral cell cluster is increased. (**i**) Summary of D-pax2 expression at late stage 11 in SOPs (orange) of the hypopharyngeal organ (Dv1,3) and the dorsal organ (Dd9,11,12). Other abbreviations are as in Figure 1.

In the early embryonic brain of vertebrates, expression of genes of the *Pax2/5/8 *subfamily is indicative for the region of the presumptive mid-hindbrain domain, which is positioned in the intervening region between the *Otx/otd *and *Hoxb1/lab *domains [4]. Therefore, I investigated the expression of two orthologous genes in *Drosophila*, *D-pax2 *and the closely related *pox-neuro *(*poxn*) [27-29]. Expression of *D-pax2 *initiates at stages 10/11 in a few cells in the truncal peripheral nervous system (most likely including the sensory organ progenitors (SOPs) of the internal and external sensory organs), and in SOPs of the antennal (Dd9,11,12), labral, and ventral hypopharyngeal-/latero-hypopharyngeal (Dv1,3) sensory organs (Figure 2a,b) [23] (see also [30,31]). At later embryonic stages the D-pax2-positive progeny of the two SOPs of the hypopharyngeal-/latero-hypopharyngeal organ are positioned lateroventrally to the foregut and, hence, are clearly separated from the brain. Both SOPs lay ventrally adjacent to the IZ of brain NBs (Figure 2b,c). In the brain, however, I have not found D-pax2 before stage 14/15 (in cells of the DC and PC) (Figure 2c), when it is likewise metamerically expressed in the ventral nerve cord [31]. These D-pax2-expressing cells in the brain and ventral nerve cord originate from pNE and NB(s), which do not express D-pax2. I therefore asked if, instead of D-pax2, the second *Pax2/5/8 *ortholog, *poxn*, is expressed in NBs of the IZ. In the trunk at stage 11, Poxn is segmentally expressed first in SOPs of the external sensory organs and slightly later in NB 2–4 (Figure 2d; see also [28]). Likewise, in the head ectoderm, Poxn is first expressed in a few cells of the developing labral and antennal appendages (which presumably contribute to the respective sensory organs) at about the same time it is found in SOPs of the truncal segments (Figure 2d,e). However, in the brain, Poxn expression was first found later (by stage 12/4) in a single cell of the DC (Figure 2f), and by stage 13 in 1–2 protocerebral cells that coexpress En and descend from NBs of the *en *head spot (hs; Figure 2g). The number of Poxn-positive cells in the DC and PC increases during embryogenesis, but importantly, all these cells develop from Poxn-negative pNE and NBs (Figure 2h).

Taken together, Poxn and D-pax2 are not expressed in brain NBs but in certain progeny cells at later embryonic stages. Despite this lack of early Poxn and D-pax2 in NBs of the IZ, I suggest the brain anlagen to be tripartite, in the sense of consisting of three spatially distinct regions: an anterior Otd domain, a posterior Lab domain and an IZ, where, at the level of the pNE and brain NBs, neither gene is expressed. In this regard it is worth noting that the D-pax2 expressing SOPs of the hypopharyngeal organ (Dv1,3) are localized immediately ventral, and the D-pax2 expressing SOPs of the dorsal organ (Dd9,11,12,13) immediately dorsal to the NBs of the IZ (Figure 2a,b,i). However, these SOPs do not contribute to the brain. Considering these findings, I propose the IZ to encompass about eight NBs of the DC (Dd1,4,5,7,8,10, Dv7,8; Figure 2i).

### Common interface of *otd *and *unpg *domains corresponds to the anterior border of the intervening zone

**Figure 3:**
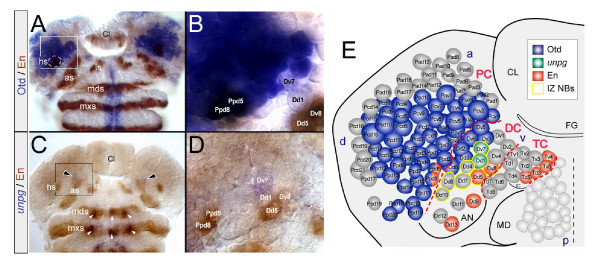
Otd and *unpg *form a common interface within the IZ. **(a,b) **Otd/En-lacZ double labelling. **(c,d) ***unpg *mRNA/En-lacZ double labelling in the peripheral head ectoderm (a,c) and deriving brain NBs at stage 11 (b,d). (b,d) Close-ups of regions framed in (a,c), respectively. (b) Note that Dd1 and Dv7 do not express Otd. (d) In the brain, *unpg *mRNA is detected in only two deutocerebral NBs (Dd1, Dv7) immediately anterior to the antennal *en *stripe (as) and bordering the Otd domain (b). **(e) **Summary of the expression of Otd, *unpg*, and En according to the colour code. Note the complementary expression of Otd and *unpg*, and that Dd1, Dv7 are part of the NBs of the IZ (IZ NBs). Red dashed lines indicate segmental borders. Other abbreviations are as in Figure 1.

*Otx2 *and *Gbx2 *are expressed in the region of the presumptive vertebrate midbrain/hindbrain domain. Their mutual repressive interaction results in a clear interface between the posterior *Gbx2 *and the adjacent anterior *Otx2 *domain, which has an important role in positioning the isthmic organizer [4]. In the late embryonic *Drosophila *brain (stage 14/15), the expression domains of the *Otx2 *ortholog, *otd*, and the *Gbx2 *ortholog, *unplugged *(*unpg*) have been reported to form a common interface at the segmental boundary between TC and DC [20]. Since in vertebrates both genes are the first factors expressed in the presumptive midbrain/hindbrain domain (from gastrulation onwards), I was interested to see if such an *otd*/*unpg *interface is established in the early anlagen of the embryonic brain, at the level of NBs. By stage 11, *unpg-lacZ *(as revealed in the enhancer trap line 1912) is expressed in the antennal and adjacent ocular pNE, as well as in most NBs in the DC and adjacent PC, as previously shown [26]. Consequently, *unpg-lacZ *and Otd are not complementarily expressed, as they exhibit an overlap within the posterior PC and anterior DC. This prompted me to investigate if the lacZ pattern reliably represents the expression of the *unpg *gene. *In situ *hybridizations showed that, in the pNE, the pattern of *unpg *transcripts does not fully match the pattern of *unpg-lacZ *(data not shown). Detectable levels of *unpg *mRNA do not become visible before stage 11 and then only in the part of the antennal ectoderm (and deutocerebral NBs) where *unpg-lacZ *is expressed strongest (Figure 3c). Accordingly, I found *unpg *mRNA only in the DC, and in a significantly smaller subset of NBs (Dd1, Dv7), immediately anterior to those deriving from the *en *antennal stripe (Figure 3d). Interestingly, Dd1 and Dv7 exactly abut the posterior limit of the domain of Otd expressing NBs (Figure 3a–c).

Taken together, procephalic Otd and *unpg *(mRNA) are complementarily expressed, exhibiting a small common interface at the anterior border of the IZ, which is positioned within the anterior half of the deutocerebral anlagen. These data suggest that the IZ and the anterior adjacent pNE, which are separated by the *otd*/*unpg *interface, represent an ancestral ectodermal territory, evolutionarily equivalent to the early embryonic vertebrate midbrain/hindbrain domain (including the *Otx2/Gbx2 *border).

### Expression of other vertebrate MHB-specific orthologs in the region around the *Drosophila otd/unpg *interface

Early in embryonic development, the region of the vertebrate MHB is characterised by the expression of several other genes, among which are *En *and *Bts1*, as well as the secreted factors *Wnt1 *and *Fgf8*. These factors have been shown to be involved in the patterning and differentiation of the evolving structures of the midbrain and anterior hindbrain [1,3,4,32]. I was interested to explore how far the expression of orthologous genes is conserved in the pNE and NBs around the *otd/unpg *interface in *Drosophila*.

**Figure 4:**
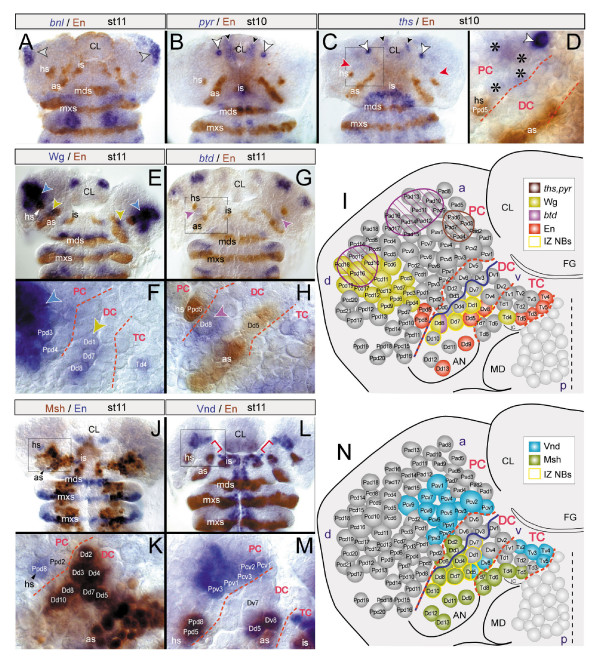
Expression of other vertebrate MHB-specific genes in the early *Drosophila *brain. Head flat preparations. **(a-d) **mRNA expression of FGF8-related genes *branchless *(*bnl*; (a); stage 11), *pyramus *(*pyr*; (b); stage 10), or *thisbe *(*ths*; (c), stage 10) in relation to Engrailed (En)-lacZ. (a) In the pNE, *bnl *transcripts are confined to most dorsal sites of the ocular pNE (arrowheads). (b) *pyr *expression is detected in the labral ectoderm (black arrowheads) and in one protocerebral NB in each hemibrain (white arrowheads). (c,d) Expression of *ths*, similar to *pyr*, is found in the labral ectoderm (black arrowheads) and in one protocerebral NB (white arrowheads). *ths *is faintly detected in a further pNE domain (red arrowheads), slightly anterior to the *en *head spot (hs) and the Ppd5 (in (d)). (d) Higher magnification of framed area in (c). Protocerebral NBs (asterisks) emanating from that area express *ths *very faintly. **(e,g,h) **Expression of Wingless protein (Wg) (e) or *buttonhead *mRNA (*btd*) (g,h) in combination with En-lacZ, or **(f) **only Wg. (e,f) Wg is expressed in the ocular (blue arrowheads) and deutocerebral pNE (brown arrowheads) and (f) in the corresponding protocerebral and deutocerebral NBs (Dd1,7,8), respectively. (g,h) *btd *is weakly expressed in a deutocerebral pNE domain ((g), purple arrowheads) and in the deutocerebral Dd8 (h) of the IZ. **(i) **Scheme summarizing the expression of Wg, *btd*, *ths*,*pyr*, and En in brain NBs at stage 11. Red dashed lines indicate neuromeric boundaries, the blue line the posterior border of the *otd *domain, which corresponds (at least partially) to the *otd/unpg *interface. Coloured hatched areas indicate neuroectodermal regions in which yet unidentified NBs express *btd *(purple), or *ths *or *pyr *(brown). **(j-n) **Expression of Muscle segment homeobox (Msh-lacZ) (j,k) or Ventral nervous system defective (Vnd) (l,m) and En (Inv in (j,k); En-lacZ in (l,m)). (j,k) The anteriomost limit of Msh expression abuts the anterior border of the deutocerebrum (DC; red line), which roughly coincides with the anterior border of the IZ. (k) Msh is expressed in dorsal NBs of the IZ (Dd4,5,7,8,10).(l,m) Vnd expression is specifically lacking in the ventral pNE (indicated by red brackets in (l)) and in all ventral NBs of the deutocerebrum (m), including Dv7 (of the IZ) at the *otd/unpg *interface (blue line in (n)). (n) Scheme summarizing the expression of Msh and Vnd in brain NBs at stage 11.

In vertebrates, *Fgf8 *is expressed in a narrow domain immediately posterior to the border between the developing mid- and hindbrain [33]. Three *Fgf8*-related genes have been described in *Drosophila*: *branchless *(*bnl*) [34], and the more closely related *pyramus *(*pyr*) and *thisbe *(*ths*) [35]. Using *in situ *hybridizations I found that, during embryogenesis, the pattern of *bnl *[34], *pyr *and *ths *transcripts [35] is the same as described previously. By stage 11, *bnl *transcripts were found at dorsal-most sites of the ocular pNE and in a few corresponding protocerebral NBs (Figure 4a). In the examined period until stage 11, detectable levels of *bnl *mRNA were not observed in the remainder of the brain anlagen. *pyr *transcripts are prominently expressed during stages 10/11 in one protocerebral NB, but are not found in the region of the *otd/unpg *interface (Figure 4b). *ths *mRNA is found in some protocerebral NBs during stages 8/9, but becomes largely downregulated by stage 10, when it is still found in low concentrations at the level of the *en *head spot. By stage 11, *ths *mRNA is restricted to a single anterior protocerebral NB (Figure 4c,d), at a position comparable to that of the *pyr *expressing NB (Figure 4b). Importantly, all procephalic *ths *expression domains lie anterior to the *otd/unpg *interface and partly within the Otd domain, which is dissimilar to the situation in vertebrates. At later embryonic stages, none of the *Fgf8*-related genes exhibit detectable levels of transcripts in the vicinity of the *otd/unpg *interface (data not shown). Hence, the expression of all three *Fgf8*-related genes in *Drosophila *is in striking contrast to the expression of *Fgf8 *at the embryonic vertebrate *Otx2/Gbx2 *border. In vertebrates, *Wnt1 *is expressed in a narrow domain just anterior to the *Otx2/Gbx2 *border and overlaps with expression of *Otx2 *[1,32]. Comparably, *Drosophila *Wingless (Wg) is coexpressed with *otd *in a protocerebral domain anterior to the *otd/unpg *interface; additionally, Wg is found in a deutocerebral domain located immediately posterior to the *otd/unpg *interface (Figure 4e,f,i) [26]. En is found in two protocerebral NBs (Ppd5,8) deriving from the *en *head spot immediately anterior to the *otd/unpg *border, and in two deutocerebral NBs (Dd5, Dv8) deriving from the antennal *en *stripe in the posterior vicinity of the *otd/unpg *border (Figure 4i,m) [25]. This is similar to the expression of *En1*,*2*, the domains of which in vertebrates span large parts of the neuroectoderm adjacent to the *Otx2/Gbx2 *border. In the zebrafish, *Bts1*, a member of the *Sp *gene family, is one of the earliest genes expressed at the presumptive midbrain/hindbrain domain [3]. In *Drosophila*, I detected transcripts of the closely related cephalic gap gene *buttonhead *(*btd*) in the same pattern as described previously [36,37]. By stage 11, *btd *mRNA was found in the dorsal antennal pNE and in one to two corresponding deutocerebral NBs located at the same AP level as the *otd/unpg *interface (Figure 4g,h,i). This again corresponds to the situation at the vertebrate MHB.

Taken together, *btd*, *en*, and *wg *are expressed in the immediate vicinity of the *otd/unpg *interface, corresponding to the expression of orthologous genes at the vertebrate *Otx2/Gbx2 *border. However, the *Fgf8*-related genes *pyr *and *bnl *are not expressed in the area of the *otd/unpg *interface, and *ths *is activated only transiently at low levels and at an improper position in relation to the *otd/unpg *interface and other MHB-specific marker genes, indicating crucial differences to the situation in the vertebrate embryo.

### Discontinuous expression of DV patterning genes *msh *and *vnd *at the *otd/unpg *interface

In the vertebrate neural tube, the order of expression of DV patterning genes of the *Nkx *and *Msx *gene families along the DV axis is analogous to that of the orthologs *ventral nervous system defective *(*vnd*) and *muscle specific homeobox *in the *Drosophila *neuroectoderm: *Nkx/vnd *are expressed in ventral regions, and *Msx/msh *in dorsal regions [38,39]. Along the AP axis, *Nkx2.2 *and *Msx3 *(which presumably represents the ancestral *Msx/msh *gene) have been reported to be discontinuously expressed at the MHB. *Nkx2.2 *exhibits a gap of expression specifically at the MHB [40]. Moreover, the anteriomost sharp limit of *Msx3 *abuts exactly the MHB [41,42]. In *Drosophila*, the AP expression patterns of Vnd and Msh exhibit striking similarities. Until stage 11, I observed a lack of Vnd expression specifically at the AP level of the *otd/unpg *interface (Figure 4l,m,n). In addition, Msh, which is expressed in the dorsal NBs of the TC and DC [25], exhibits an anterior limit that largely coincides with the AP level of the *otd/unpg *interface (Figure 4j,k,n). Thus, the discontinuous expression of Vnd and Msh at the *otd/unpg *interface is similar to *Nkx2.2 *and *Msx3 *at the *Otx2/Gbx2 *border. This lends further support to the proposed ancient evolutionary origin of the pNE anteriorly and posteriorly adjacent to the *otd/unpg *interface.

## Discussion

This comprehensive expression analysis of factors orthologous to key regulatory genes of the embryonic vertebrate MHB was aimed at clarifying whether the early embryonic brain anlagen in *Drosophila *reveal a tripartite regionalization, contain a conserved *Otx/Gbx *border and include an ectodermal territory that shares similarities with the anlagen of the vertebrate MHB. I have focussed my study mainly on the early phase of embryonic brain development, because positioning and establishment of the MHB region is a very early decision in vertebrate central nervous system (CNS) development (taking place in the neuroectoderm before and during the formation of NBs). In addition, in *Drosophila*, the segmental organization of the brain is most clearly displayed in this phase and the examination can be done at the highest resolution, at the level of individually identifiable NBs.

### Is a tripartite regionalization of the anterior CNS, based on *Otx, Pax2/5/8*, and *Hox1 *orthologous domains, conserved in bilaterians?

**Figure 5:**
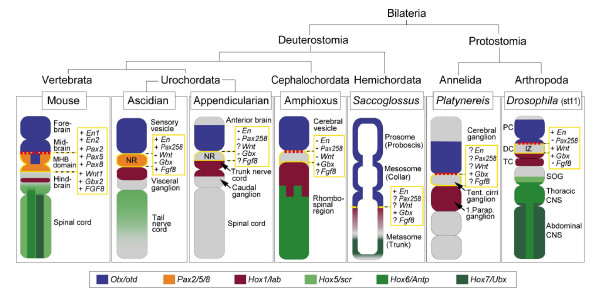
Comparison of CNS regions and gene expression domains among chordates, hemichordates, annelids, and arthropods. Focus is on the anteroposterior regionalization of the anterior CNS, including the brain. The schemes reflect the situation in the CNS of the vertebrate mouse (at about embryonic day 10–12.5), the ascidians *Ciona *and *Halocynthia *[11, 13, 43, 44, 54], the appendicularian *Oikopleura *(at the late hatchling stage) [14], the amphioxus *Branchiostoma *(at the 10–13 somite stages) [15, 16, 45, 76], the hemichordate *Saccoglossus *(at the one gill slit stage) [48, 49], the polychaete annelid *Platynereis *(neuroectoderm at the metatrochophora stage) [46, 47], and the arthropod *Drosophila *(at stage 11, st11; this study). Expression of *Otx/otd*, *Pax2*,*5*,*8*, *Hox1/lab*, *Hox5/scr*, *Hox6/Antp*, and *Hox7/Ubx *genes is indicated according to the colour code. The dashed line in red indicates the interface between *Otx/otd *and *Gbx/unpg *expression domains. The expression of further genes within the gap (encircled in yellow) between the anterior *Otx/otd *and posterior *Hox1/lab *domains is noted: '+' indicates expression of the respective gene; '-' absence of expression; and '?' expression is not yet determined. The phylogenetic tree is based on [76]. For further details, see the text. IZ, intervening zone; MHB domain, midbrain/hindbrain boundary domain; NR, neck region; Parap., Parapodial; Tent., Tentacular.

Deuterostomes comprise the hemichordate/echinoderm clade and the chordates, which include vertebrates and the closely related invertebrates, amphioxus (cephalochordates) and tunicates (urochordates) (Figure 5). On the basis of the expression of highly conserved regulatory genes, the anterior part of the early vertebrate CNS is considered to exhibit a fundamentally tripartite regionalization along the AP axis: *Otx *is expressed in the forebrain/midbrain, *Hox *genes in the hindbrain/spinal cord, and *Pax2/5/8 *genes in the intervening domain that constitutes the MHB region [1] (Figure 5). This tripartite organization of gene expression is also found in ascidian tunicates, that is, in *Halocynthia *and *Ciona*. Tunicates, together with amphioxus, represent the closest relatives of vertebrates [43]. Expression of a *Pax2/5/8 *ortholog has been reported for the ascidian neck region between the *Otx *and *Hox1 *domains [11,44] (Figure 5), leading to the speculation that the neck region is orthologous to the vertebrate MHB [11,19]. This is supported by the findings that *Pax2/5/8 *is also expressed in the neck region of *Ciona savingyi *[45], and *Pax2/5/8*, *Fgf8/17/18*, and *En *in *Ciona intestinalis *[13]. However, accumulating data show that the expression of MHB-specific markers is already diverse among the tunicates. In contrast to the ascidians, for example, the expression of *Pax2/5/8 *in the appendicularian tunicates (that is, in *Oikopleura*) is lacking in the neck [14]. Furthermore, whereas in *C. savignyi En *is found in the *Pax2/5/8 *expressing neck region [45], in *C. intestinalis En *is expressed in two domains, anteriorly and posteriorly adjacent to the *Pax2/5/8 *expressing neck [13]. *Wnt *orthologous genes seem to be generally lost in the ascidian genome [46,47] and, therefore, are not expressed in the neck region. Since it has been difficult to deduce the ancestral pattern, the existence of a MHB homologous region in tunicates is controversial [14,48]. In the intervening region of the protochordate amphioxus, expression of *Pax2/5/8 *as well as of *Wnt1 *and *En *is lacking, similar to the situation in the appendicularian tunicate *Oikopleura *[15-17] (Figure 5). However, a tripartite organization seems basically conserved, considering the existence of an anterior *Otx *domain, a posterior *Hox1 *domain, and an intervening region in which neither of these genes is expressed [16].

The situation in amphioxus and *Oikopleura *is comparable to the findings made in *Drosophila *in this study (Figure 5). The early embryonic brain can be subdivided into an anterior domain of *Otx/otd *expression, encompassing most of the PC and an adjacent part of the DC, and a posterior domain of *Hox1/lab *expression, encompassing the TC. Both domains are separated by an IZ, covering part of the deutocerebral pNE and eight deutocerebral NBs, in which neither gene is expressed. The two *Pax2 *genes (*D-pax2 *and *poxn*) are not expressed in the early embryonic IZ, which is a significant difference to the situation in vertebrates and ascidian tunicates (*Halocynthia *and *Ciona*). However, both *Pax2 *genes are expressed during later embryonic brain development [20], when they are also expressed in segmentally repetitive neuronal clusters in the VNC. Importantly, at that time, AP patterning (that is, segmentation) and early neurogenesis (that is, formation/specification of NBs) are already completed. Thus, the later phase of expression of both genes is presumably involved in specification/differentiation of neural progeny cells, but is not compatible with an early function in patterning or specification of the pNE or NBs at the IZ. Accordingly, no obvious brain phenotype has been observed in *poxn *or *D-pax2 *mutants [20]. On the other hand, early expression of *poxn *and *D-pax2 *is found in progenitors of the peripheral nervous system, some of which are placed in immediate vicinity of NBs of the IZ, suggesting an early function of both genes in the development of head sensory structures. This is in accordance with findings made in the trunk, where *poxn *and *D-pax2 *are first expressed in the precursors of the developing peripheral nervous system [28,31] (Figure 2a,d), and later on in the ventral nerve cord.

Similarly, in the neuroectoderm of another protostomia, the polychaete annelid *Platynereis dumerilii*, an anterior *Otx *domain [49] seems to be spatially separated from a posterior *Hox1 *domain (Figure 5) [50]; although expression of a Pax2/5/8 ortholog has been reported in the trunk nerve cord [51], it has not yet been described for the brain. Hemichordates, distant deuterostomes, which do not have an internalized CNS but a body-encircling basiepithelial nerve net, reveal an anterior *Otx *and a posterior *Hox1 *expression domain, comparable to the situation in chordates, *Platynereis*, and *Drosophila *(Figure 5). A *Poxn *ortholog has been identified in the hemichordate *Saccoglossus kowalevskii*, but it is distinct from the *Pax2/5/8 *group of genes; expression of *Pax2/5/8 *orthologs has not yet been characterized [52]. Lastly, the MHB-specific marker *En *is expressed in the intervening region between the *Otx *and *Hox1 *domains [53].

Taken together, the data suggest that a tripartite ground plan characterizing the development of the chordate (and perhaps polycheate and hemichordate) brain is basically also present in the insect brain, which is in agreement with Hirth *et al*. [20]. However, in the early embryonic *Drosophila *brain expression of *Pax2/5/8 *orthologs is absent in the IZ. Nevertheless, the presence of a brain region that expresses neither *otd *nor *labial *indicates that a domain regionally homologous to the vertebrate MHB domain may also exist in *Drosophila *(see also the following discussion). Thus, it is tempting to speculate that a tripartite ground pattern (which lacks early *Pax2/5/8 *expression) was acquired already in the bilaterian ancestor.

### Is the *Otx/Gbx *border an ancestral condition in bilaterians?

In vertebrates,*Otx2 *and *Gbx2 *are among the earliest genes expressed in the nervous system [33,54]. The establishment of a border between the complementary neuroectodermal domains of *Otx2 *(anterior) and *Gbx2 *(posterior) is the initial step in MHB development. The border, which forms due to mutual repression of these genes, is crucial for the proper positioning of the MHB [1,2]. In the early amphioxus embryo, *Gbx *and *Otx *domains abut the cerebral vesicle and hindbrain; therefore, this border is likely to be homologous to the vertebrate MHB (although it seems unlikely that this boundary has organizer properties) [48]. Yet there are no *Gbx *genes in urochordates, suggesting that they have been lost secondarily in this lineage [14]. In hemichordates by contrast, the domains of *Otx *and *Gbx *overlap considerably, indicating that these genes do not antagonize each other [53]. This is reminiscent of the initial phase of *Otx *and *Gbx *expression in amphioxus, when the domains of both genes slightly overlap, although they sharply abut later on [48].

Similar to the situation in vertebrates and amphioxus, in *Drosophila *a common border can be recognized between the procephalic expression domains of *otd *and *unpg *(the *Gbx2 *ortholog). However, expression of *unpg *initiates significantly later (by stage 11) than that of *otd *(by stage 6), different to the situation in vertebrates (Additional file 1). As shown at the level of NBs, the common border between both expression domains is positioned in the anterior DC. Thus, the *otd/unpg *interface is located more anterior and does not coincide with the boundary between the TC and DC as supposed previously [20]. Nevertheless, in the late embryonic brain, there is evidence that *otd *and *unpg *negatively regulate each other, similar to the genetic interaction of their vertebrate orthologs [20]. In this regard it is important to note that in the anterior neuroectoderm of the polychaete *Platynereis*, a boundary between the anterior domain of *Otx *expression and a posterior domain of *Gbx *expression has also been reported recently [49], supporting its ancient origin.

In summary, the border between the *Otx/otd *and *Gbx/unpg *domains found in chordates, polychaetes, and *Drosophila *(but not in hemichordates) suggests a homologous use of both genes in AP patterning. The *Otx2/Gbx2 *border in vertebrates determines the position of the MHB. Considering that the *otd/unpg *border can be identified in the early embryonic brain, and that the genetic interactions of *otd *and *unpg *appear to be conserved [20], it seems likely that at least the machinery for positioning the MHB might already be existent in *Drosophila*. Therefore, the border between the *Otx/otd *and *Gbx/unpg *domains may represent an ancestral condition in bilaterians.

### Does the pNE surrounding the *otd/unpg *interface share homology with the vertebrate MHB organizer?

**Figure 6:**
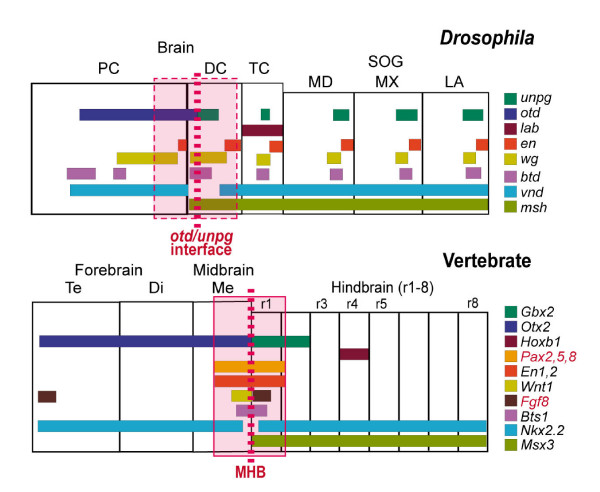
Comparison of brain organization and expression of key developmental genes involved in CNS regionalization and MHB formation in vertebrates and *Drosophila*. Data for *Drosophila *reflect the situation at stage 11, data for vertebrates reflect the situation in mouse at about embryonic day 10 (E10; see [1]; *Nkx2.2*, [40]; *Msx3*, [41]; the data for *Bts1 *are from a comparable developmental stage in zebrafish [3]). The boxed area coloured in light red indicates the neuroectodermal region around the MHB in vertebrates, according to the expression domains of *Pax2*,*5 *and *8 *and *En1 *and *2*. Although early *Pax2/5/8 *gene expression is lacking in *Drosophila*, the corresponding box may indicate a similar domain around the *otd/unpg *border. Identical colours indicate orthologous genes. Vertebrate gene names in red indicate those genes that are not or differently expressed at the *Drosophila otd/unpg *interface. For details, see the text. PC, DC, TC; proto-, deuto-, tritocerebrum; MD, MX, LA, mandibular, maxillary, labial neuromere, respectively; SOG, subesophageal ganglion. Di, Me, Te, Dien-, Mesen-, Telencephalon, respectively; r1–8, rhombomeres 1–8.

After the *Otx2/Gbx2 *border in vertebrates has been formed at the prospective MHB, several other key genetic factors, such as *Pax2*, *En*, *Wnt1*, and *Fgf8 *are expressed in an ordered spatial and temporal mode [1,2,4,5]. A comparison of the spatial and temporal expression of those gene orthologs at the vertebrate MHB and the *Drosophila otd/unpg *interface is given in Figure 6 and Additional file 1, respectively. As discussed above, the formation of the *otd/unpg *interface within the IZ already indicates that the corresponding neuroectodermal domain in *Drosophila *might share basic similarities with the vertebrate MHB. Moreover, comparable to the situation in vertebrates, *en *is expressed at the *otd/unpg *border, in a deutocerebral and in a protocerebral domain. However, in contrast to vertebrates [55,56], early expression of *en *cannot be activated by *Pax2 *genes in *Drosophila *since the latter are not expressed in the early brain. Nevertheless, from embryonic stage 13 onwards, some of the protocerebral *en *cells coexpress *poxn*, suggesting possible genetic interactions at those later stages (Figure 2g,h); interestingly, coexpression of both genes is not observed elsewhere in the CNS. In *Ciona*, two *En *domains have been recognized to be positioned similarly to those in *Drosophila*, in the immediate vicinity of the neck region [13]. Since neither of the *En *domains coexpresses *Pax2/5/8*, both in *Drosophila *and *Ciona*, *Pax2/5/8 *is unlikely to activate *En *expression.

As shown above, the expression data for other conserved key factors, such as *wg*,*btd*, and the DV patterning genes *msh *and *vnd*, suggest that the pNE surrounding the *otd/unpg *interface may represent an ancestral territory that shares molecular similarities with the region around the vertebrate MHB. Important differences are that, in *Drosophila*, expression of *Fgf8*-related genes, *bnl *and *pyr*, and early expression of *Pax2/5/8 *is not found at the *otd/unpg *interface. In agreement with that, no obvious brain phenotype has been observed in *bnl *mutant embryos [20]. Only *ths *expression is detected, but it is very faint, transient, and anterior to the *otd/unpg *interface. Since *ths *seems to be colocalized with *wg *expression in this region, it is unlikely to serve the same function as *Fgf8 *at the vertebrate MHB. These differences in the expression of *Fgf8*-like genes in *Drosophila *are most significant, as *Fgf8 *is the key player at the vertebrate MHB, eliciting the expression of other organizer genes and exerting most of the organizer functions [2].

In addition, the temporal gene expression profile of this brain territory and the vertebrate MHB domain reveals distinctions (Additional file 1); for example, *unpg *is expressed significantly later than *otd*. Altogether, the spatial and temporal dissimilarities indicate that during the early period of neurogenesis, basic regulatory interactions among these genes, crucial to exert organizer properties, do not exist or are modified. In the light of these data, it is therefore very unlikely that the pNE surrounding the *otd/unpg *interface represents a functional homolog of the MHB organizer. This supports the assumption (see work on *Amphioxus *[48]) that during evolution the *Otx/Gbx *border was established before it became equipped with organizer function. Since there is also no convincing evidence for an MHB organizer in any tunicate, it presumably first evolved in the vertebrate lineage.

### Equivalents of 'hindbrain' and 'spinal cord' in *Drosophila*?

In the vertebrate neural tube, the border between hindbrain and spinal cord is supposed to be approximately indicated by the anterior limit of *Hoxb5 *expression [57-59]. This molecular regionalization may be conserved among chordates, since in the tunicates *Ciona *and *Oikopleura *the restricted expression of a *Hox5 *ortholog is also suggested to coincide with the anterior border of the spinal chord [14,19,60,61] (Figure 5). However, recent data in vertebrates [59,62-64] show that the *Hoxb5 *domain, although initially confined to the spinal cord, extends rostrally into the developing posterior hindbrain. Unlike *Hoxb5*, the rostral borders of *Hoxb6 *and *Hoxb7 *domains finally come to lie close to the transition between hindbrain and spinal cord [65], and might, therefore, be more suitable indicators to distinguish between both CNS domains. In *Drosophila*, the anterior border of the *Scr/Hoxb5 *domain maps within the maxillary neuromere, those of *Antp/Hoxb6 *and *Ubx/Hoxb7 *domains within the labial and third thoracic neuromere, respectively (Additional file 2). Assuming that, in analogy to vertebrates [66], the posterior border of *otd *expression (within the anterior DC) sets the anterior limit, the hindbrain equivalent would comprise four neuromeres, when considering the anterior border of Scr expression as its posterior limit, or, what is more likely, up to eight neuromeres, when considering the anterior Ubx expression border as the posterior limit. Interestingly, a comparable number of seven to eight rhombomeres comprises the vertebrate hindbrain [67]. In this perspective, the CNS region posterior to the anterior limit of the Scr or Ubx domain would be equivalent to the vertebrate spinal cord.

The vertebrate hindbrain is clearly subdivided into segmental units [67], as opposed to the mid- and forebrain in which the underlying metamerism is unclear [40,68,69]. Accordingly, the segmented part of the anterior CNS is separated from the less overt segmented part by the *Otx2/Gbx2 *border. The situation in *Drosophila *exhibits similarities. Whereas the segmental characteristics of DC, TC and ventral nerve cord are obvious, they are cryptic in the PC [26,25]. Considering the position of the *otd/unpg *border within the anterior DC, this border, as in vertebrates, separates the segmented part of the CNS (including the posterior DC, the TC, the gnathal, thoracic and abdominal CNS) from an anterior part (the PC) in which the metameric identity is less obvious.

## Conclusion

Molecular characterization of the neuroectoderm and of individually identified neural stem cells in the early *Drosophila *embryo, indicate the existence of a non-segmental *Otx/Gbx *orthologous interface located within the anterior DC. Furthermore, my data support the idea that the area surrounding this interface (encompassing the anterior DC/posterior PC) may represent an ancestral territory that shares molecular similarities with the region around the vertebrate MHB. Otherwise, lack of expression of *Pax2/5/8 *and *Fgf8*-related genes, and significant differences in the expression onset of other key regulators at the *otd/unpg *interface, imply that genetic interactions crucial to exert vertebrate organizer activity do not exist or are modified in the early embryonic brain of *Drosophila*.

## Materials and methods

### *Drosophila *strains

The following fly strains were used: Oregon R (wild type), *unplugged-lacZ *[22], *engrailed-lacZ *[70].

### Staging and mounting of embryos

Staging of the embryos was done according to [71]; additionally, the trunk NB pattern [22] was used as a further reference for staging. Flat preparations of the head ectoderm of stained embryos and mounting were done as described previously [23].

### Antibodies and immunohistochemistry

Embryos were dechorionated, fixed and immunostained according to previously published protocols [72]. The following primary antibodies were used: mouse-anti-Antennapedia (1:20; DSHB, Iowa City, IA, USA), rabbit-anti-Atonal (1:5000; A Jarman, Edinburgh, UK), anti-DIG-AP (1:1,000; Roche, Mannheim, Germany), mouse-anti-Engrailed/Invected (1:4; DSHB), mouse-anti-β-galactosidase (1:500; Promega, Madison, WI, USA), rabbit-anti-β-galactosidase (1:2,500; Cappel, Costa Mesa, CA, USA), rat-anti-Labial (F Hirth, London, UK), rabbit-anti-Muscle specific homeobox (1:500; MP Scott, Palo Alto, CA, USA), rabbit-anti-Pax2 (1:50; M Noll, Zürich, Switzerland), mouse-anti-Pox-neuro (1:100; C Dambly-Chaudiere, Montpellier, France), rabbit-anti-Sex comb reduced (1:1,000, T Kaufman, Bloomington, IN, USA), mouse-anti-Ultrabithorax (1:20; DSHB), rabbit-anti-Ventral nervous system defective (1:500; CQ Doe, Eugene, OR, USA), mouse-anti-Wingless (1:10; DSHB). The secondary antibodies (all from Dianova, Hamburg, Germany) were either biotinylated (goat anti-mouse, goat anti-rabbit) or alkaline phosphatase-conjugated (goat anti-mouse, goat anti-rabbit, goat anti-rat) and diluted 1:500.

### Whole mount *in situ *hybridization

Dioxigenin (DIG)-labelled *buttonhead *RNA probe was synthesized using *Hin*dIII linearised pBKS-*btd *[73] as a template with T7 polymerase. Other DIG-labelled RNA probes were synthesized using oligonucleotide primers amplified by PCR on wild-type genomic DNA: *branchless *(1,209 bp; forward primer CAGAACTACAACACTTACTCCTCC, reverse primer CTCGTAGCTCGCATCTTCTAGG); *pyramus *(2,020 bp, forward primer GGCAATCAGAACTTTAGTAGCG, reverse primer CAGACCACCATCGTTATGATTC); *thisbe *(2,284 bp, forward primer GCCCAATGTCAGCCACATCGG, reverse primer GTCGAGGTGGGCAGGAACC); *unplugged *(908 bp, forward primer GTGTCTGCTCGGGAACA-GAAACG, reverse primer GTCCATCTCGCCGTTGTAGTTCC). In all cases, the resulting PCR fragment was used as a template. All DIG-labelled RNA probes were prepared using a DIG-RNA-labelling mix (Roche) according to the manufacturer's protocol. The hybridization on embryos was performed as described previously [74,75].

### Documentation

Embryos were viewed under a Zeiss Axioplan equipped with Nomarski optics using 40×, 63× and 100× oil immersion objectives. Pictures were digitized with a CCD camera (Contron progress 3012) and different focal planes were combined using Adobe Photoshop 7.0. Semi-schematic presentations are based on camera lucida drawings.

## Competing interests

The author(s) declare that they have no competing interests.

## Supplementary Material

Additional File 1 Expression onset of MHB-specific key developmental genes in three different vertebrate species compared to orthologous factors around the *otd/unpg *interface in *Drosophila*. In vertebrates, the temporal order of genetic interactions among MHB-specific genes is closely reflected by the temporal order of the onset of their expression. *Otx2 *and *Gbx2 *are the first genes expressed (from gastrulation onwards), succeeded by the expression of further genes in the following order: *Bts1 *[3], *Pax2*, *En1/2*, *Wnt1 *and *FGF8 *[4]. In *Drosophila*, comparable to vertebrates, *otd *is the first gene expressed at the presumptive region of the *otd/unpg *interface, already before gastrulation (stage 6). In contrast, *unpg *is expressed significantly later than *otd*, not before stage 11, when it is also found in the TC and in a segmental pattern in the ventral nerve cord. Similar to *otd*, the head gap gene *btd *is expressed before gastrulation; in the pNE and NBs at the later *otd/unpg *interface it is not detected before stages 7/8. Furthermore, *wg *and *en *are expressed in the region at about stage 8, thus later than *otd *and *btd*, but before *unpg*. This indicates that head segments can be distinguished already before *unpg *expression initiates. In contrast to vertebrates, *D-pax2 *and *poxn *are expressed significantly later than *en*, but neither in the pNE nor in brain NBs (indicated by the stippled line for *poxn*). Moreover, expression of the *Fgf8*-related genes *bnl *and *pyr *is lacking around the *otd/unpg *interface. Transient expression of *ths *(indicated by stippled line), is unlikely to reflect MHB-specific expression of *Fgf8*. The temporal onset of gene expression is in part reminiscent of the situation in vertebrates (for example, of *otd*,*btd*,*en*,*wg*), but otherwise discloses significant differences (for example, *unpg*,*D-pax2*,*poxn*,*Fgf8*-related genes), implying that the chronological order of possible genetic interactions at the *otd/unpg *interface seems to be different from those at the vertebrate MHB. Data for mouse, zebrafish and chick are according to Figure 1 in [4] and references therein. Mouse: HDF, head fold stage; s, somite stage. Zebrafish: tb, tail bud period; s, segmentation period; h, hatching period. Chick: HH, stages after Hamburger and Hamilton. n.e., not expressed in the investigated period until stage 13; n.d., not determined. Click here for file

Additional File 2 Mapping of a 'hindbrain-like' CNS domain in *Drosophila*. Expression of **(a,b) **Scr, **(c,d) **Antp, or **(e,f) **Ubx in combination with En-lacZ (En) at late stage 11. (b,d,f) Close-ups of the domains boxed in (a,c,e) at the level of NBs. Scr, Antp, and Ubx are parasegmentally expressed. (a,b) The anterior limit of the Scr expression coincides with the anterior border of the maxillary *en *stripe (mxs); the Scr domain covers the posterior compartment of the maxillary and the anterior compartment of the labial neuromere (see also [77]). (c,d) The anterior limit of the Antp domain coincides with the anterior border of the labial *en *stripe (las); the Antp domain covers the posterior compartment of the labial, all thoracic, as well as weakly all abdominal neuromeres (see also [78,79]). (e, f) The anterior limit of the Ubx domain corresponds to the anterior border of the *en *stripe in the third thoracic neuromere (t3s); Ubx is found in the posterior compartment of the third thoracic neuromere and in the abdominal neuromeres 1–8. **(g) **Model of the extension of a hindbrain-like CNS domain in *Drosophila *(corresponding NBs are encircled in grey). The expression domains of En, Otd, Scr, as well as the anterior domains of Antp and Ubx are indicated. Additionally, the SOPs of the dorsal organ and hypopharyngeal/latero-hypopharyngeal organ are indicated. For further details, see the text. T1–T3, first to third thoracic neuromeres; A1, first abdominal neuromere. Other abbreviations are as in the figures. Click here for file
