# Supplementary figures and images for: A procephalic territory in Drosophila exhibiting similarities and dissimilarities compared to the vertebrate midbrain/hindbrain boundary region
Source: Neural Dev. 2007 Nov 5;2:23. doi: 10.1186/1749-8104-2-23 (PMC2206033; doi:10.1186/1749-8104-2-23)

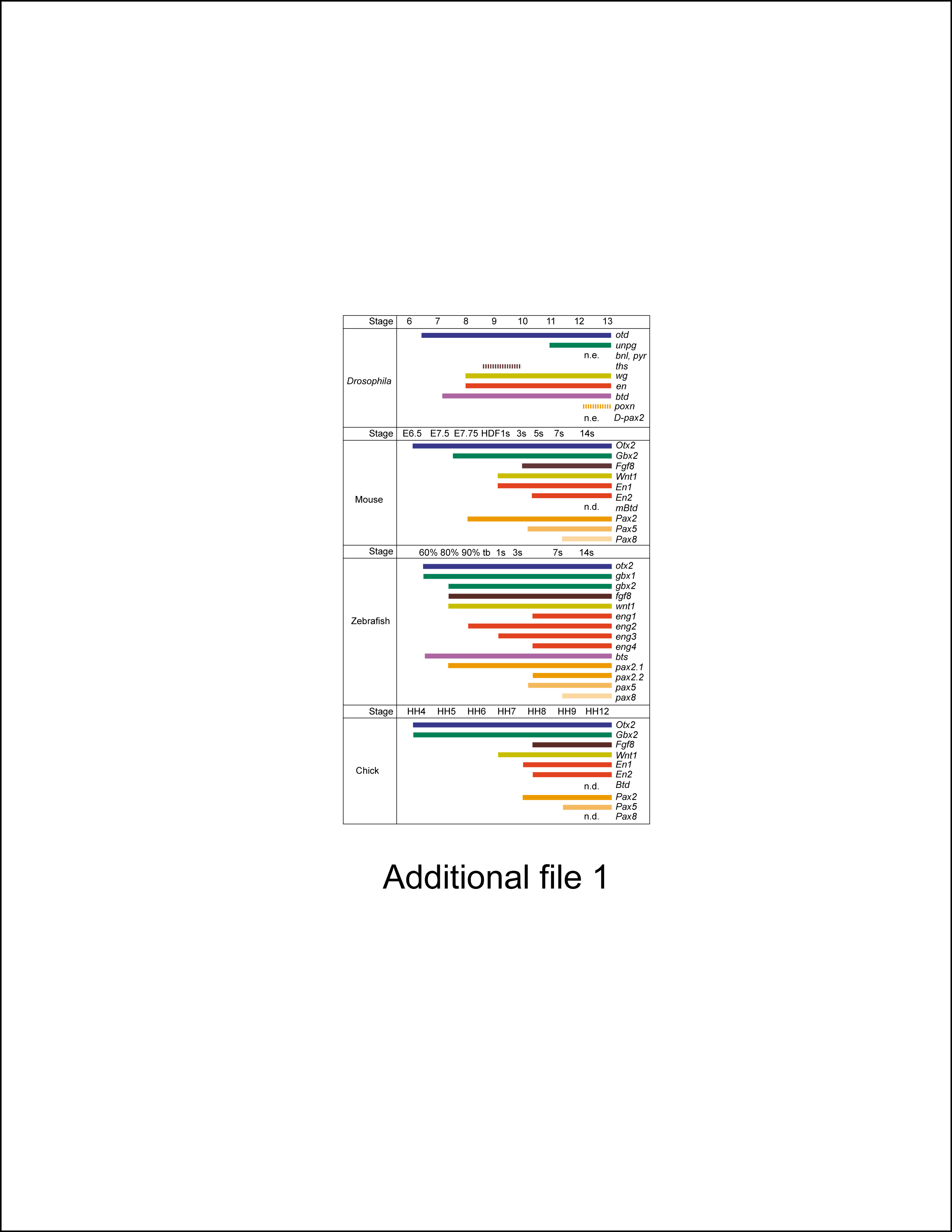

Supplement: Additional File 1 — Expression onset of MHB-specific key developmental genes in three different vertebrate species compared to orthologous factors around the otd/unpg interface in Drosophila. In vertebrates, the temporal order of genetic interactions among MHB-specific genes is closely reflected by the temporal order of the onset of their expression. Otx2 and Gbx2 are the first genes expressed (from gastrulation onwards), succeeded by the expression of further genes in the following order: Bts1 [3], Pax2, En1/2, Wnt1 and FGF8 [4]. In Drosophila, comparable to vertebrates, otd is the first gene expressed at the presumptive region of the otd/unpg interface, already before gastrulation (stage 6). In contrast, unpg is expressed significantly later than otd, not before stage 11, when it is also found in the TC and in a segmental pattern in the ventral nerve cord. Similar to otd, the head gap gene btd is expressed before gastrulation; in the pNE and NBs at the later otd/unpg interface it is not detected before stages 7/8. Furthermore, wg and en are expressed in the region at about stage 8, thus later than otd and btd, but before unpg. This indicates that head segments can be distinguished already before unpg expression initiates. In contrast to vertebrates, D-pax2 and poxn are expressed significantly later than en, but neither in the pNE nor in brain NBs (indicated by the stippled line for poxn). Moreover, expression of the Fgf8-related genes bnl and pyr is lacking around the otd/unpg interface. Transient expression of ths (indicated by stippled line), is unlikely to reflect MHB-specific expression of Fgf8. The temporal onset of gene expression is in part reminiscent of the situation in vertebrates (for example, of otd,btd,en,wg), but otherwise discloses significant differences (for example, unpg,D-pax2,poxn,Fgf8-related genes), implying that the chronological order of possible genetic interactions at the otd/unpg interface seems to be different from those at the vertebrate MHB. Data for mou [file 1749-8104-2-23-S1.jpeg]

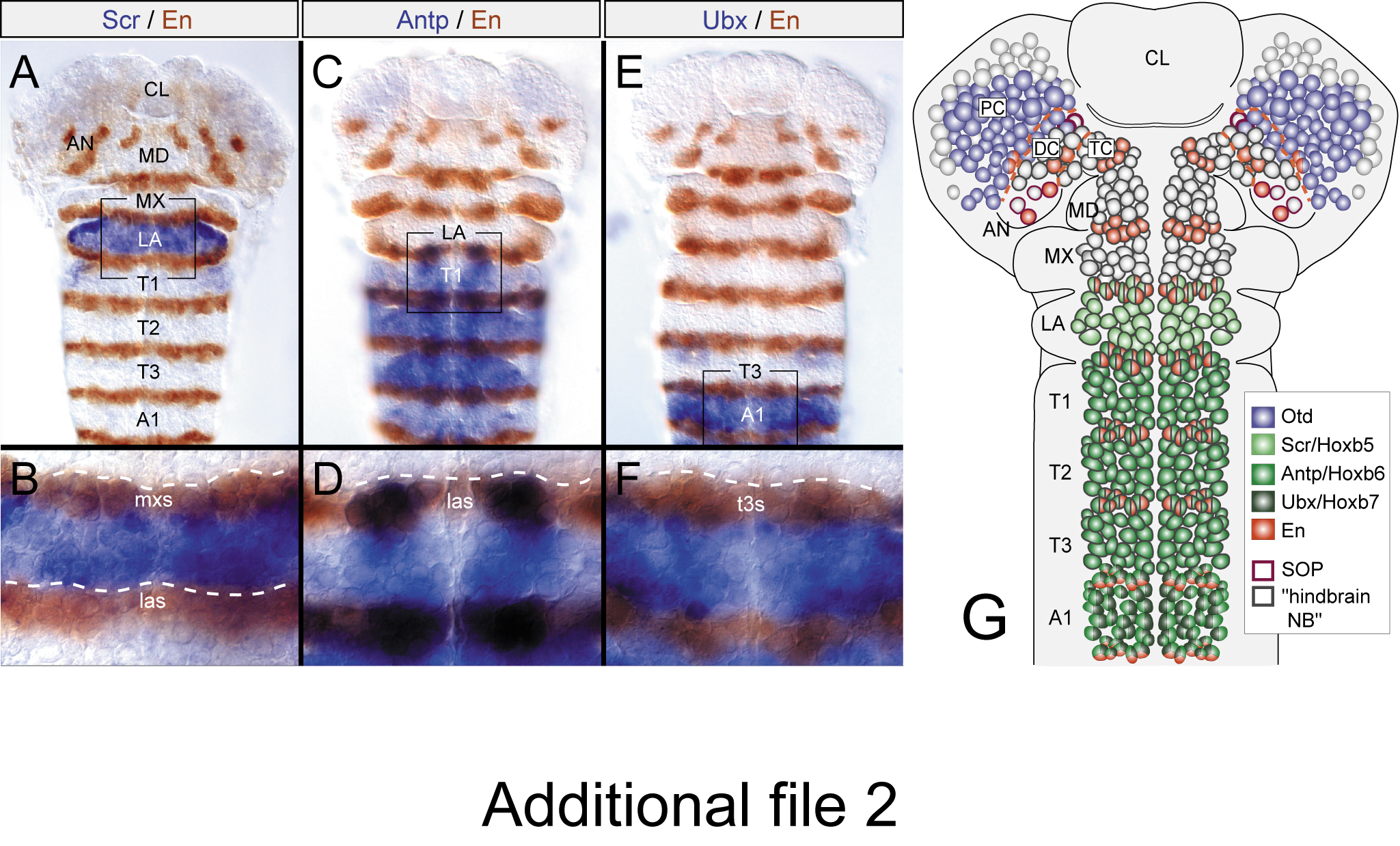

Supplement: Additional File 2 — Mapping of a 'hindbrain-like' CNS domain in Drosophila. Expression of (a,b) Scr, (c,d) Antp, or (e,f) Ubx in combination with En-lacZ (En) at late stage 11. (b,d,f) Close-ups of the domains boxed in (a,c,e) at the level of NBs. Scr, Antp, and Ubx are parasegmentally expressed. (a,b) The anterior limit of the Scr expression coincides with the anterior border of the maxillary en stripe (mxs); the Scr domain covers the posterior compartment of the maxillary and the anterior compartment of the labial neuromere (see also [77]). (c,d) The anterior limit of the Antp domain coincides with the anterior border of the labial en stripe (las); the Antp domain covers the posterior compartment of the labial, all thoracic, as well as weakly all abdominal neuromeres (see also [78,79]). (e, f) The anterior limit of the Ubx domain corresponds to the anterior border of the en stripe in the third thoracic neuromere (t3s); Ubx is found in the posterior compartment of the third thoracic neuromere and in the abdominal neuromeres 1–8. (g) Model of the extension of a hindbrain-like CNS domain in Drosophila (corresponding NBs are encircled in grey). The expression domains of En, Otd, Scr, as well as the anterior domains of Antp and Ubx are indicated. Additionally, the SOPs of the dorsal organ and hypopharyngeal/latero-hypopharyngeal organ are indicated. For further details, see the text. T1–T3, first to third thoracic neuromeres; A1, first abdominal neuromere. Other abbreviations are as in the figures. [file 1749-8104-2-23-S2.jpeg]
